# Crystal structure of 4,4′-(disulfanedi­yl)dipyridinium chloride triiodide

**DOI:** 10.1107/S2056989024004213

**Published:** 2024-05-21

**Authors:** Enrico Podda, M. Carla Aragoni, Claudia Caltagirone, Greta De Filippo, Alessandra Garau, Vito Lippolis, Annalisa Mancini, Anna Pintus, James B. Orton, Simon J. Coles, Massimiliano Arca

**Affiliations:** a Centro Servizi di Ateneo per la Ricerca (CeSAR), Università degli Studi di Cagliari, S.S. 554 bivio Sestu, Monserrato, 09042 Cagliari, Italy; b Dipartimento di Scienze Chimiche e Geologiche, Università degli Studi di Cagliari, S.S. 554 bivio Sestu, Monserrato, 09042 Cagliari, Italy; c UK National Crystallography Service, School of Chemistry, Faculty of Engineering and Physical Sciences, University of Southampton, Southampton SO17 1BJ, United Kingdom; Universidade Federal do ABC, Brazil

**Keywords:** bis­(pyridine-4-yl)di­sulfide, polypyridyl donors, halogen bonding, polyhalides, SC-XRD, crystal structure

## Abstract

4,4′-(Disulfanedi­yl)dipyridinium chloride triiodide, C_10_H_10_N_2_S_2_^2+^·Cl^−^·I_3_^−^, (**1**) was synthesized by reaction of 4,4′-di­pyridyl­disulfide with ICl in a 1:1 molar ratio in di­chloro­methane solution. The structural characterization of **1** by SC-XRD analysis was supported by elemental analysis, FT–IR, and FT–Raman spectroscopic measurements.

## Abstract 2

4,4′-Disulfanediyldipyridinium chloride triiodide (**1**) was synthesized. The structural characterization of **1** by SC-XRD analysis was supported by elemental analysis, FT–IR, and FT–Raman spectroscopic measurements.

## 1. Chemical context

The reactions of pnictogen/chalcogen donors with dihalogens X_2_ or inter­halogens XY (X, Y = Cl, Br, I) afford a variety of products depending on the nature of the donor, the dihalogen/inter­halogen, and the reaction conditions (Aragoni *et al.*, 2008 ▸; Rimmer *et al.*, 1998 ▸; Aragoni *et al.*, 2022 ▸; Knight *et al.*, 2012 ▸). For chalcogen donors, charge–transfer (CT) ‘spoke’ adducts, hypercoordinate ‘T-shaped’ adducts, halonium adducts, and different types of cationic oxidation products of the donors have been identified and structurally characterized (Knight *et al.*, 2012 ▸; Saab *et al.*, 2022 ▸). Worthy of note, diiodine CT-adducts have been extensively investigated, also with a view to their application as leaching agents for toxic (Isaia *et al.*, 2011 ▸) and precious metals (Zupanc *et al.*, 2022 ▸) in waste from electrical and electronic equipment (WEEE). Among the pnictogen donors, many studies have focused on (poly)pyridyl derivatives. Analogous to S/Se-donors, the reactions of pyridyl donors with X_2_/XY have resulted in the formation of CT-adducts featuring a linear N⋯X—Y group (Kukkonen *et al.*, 2019 ▸; Tuikka & Haukka, 2015 ▸) and halonium derivatives with an N⋯X^+^⋯N moiety (X = I; Y = Cl, Br, I) (Kukkonen *et al.*, 2019 ▸; Batsanov *et al.*, 2005 ▸, 2006 ▸). In addition, N-protonated pyridinium cations were obtained, whose charge can be counterbalanced by discrete halides or extended fascinating networks (Aragoni *et al.*, 2004 ▸; Aragoni *et al.*, 2023 ▸). Oxidation of the aromatic heterocycle to give a cationic radical species followed by solvolysis or reaction with incipient moisture has been proposed as a possible explanation for the formation of pyridinium cations (Rimmer *et al.*, 1998 ▸; Aragoni *et al.*, 2023 ▸).

The nature of the products isolated in the solid state is reflected in their peculiar FT–Raman response (Aragoni *et al.*, 2004 ▸, 2008 ▸; Pandeeswaran *et al.*, 2009 ▸). In particular, an elongation of the perturbed X–Y moiety with respect to the free halogen/inter­halogen is found in CT-adducts, which determines a low energy shift of the relevant Raman-active stretching vibration (Aragoni *et al.*, 2008 ▸). When polyhalide networks are formed, the stretching vibrations of the inter­acting synthons can be detected in the low-energy region of the FT–Raman spectrum (Aragoni *et al.*, 2008 ▸, 2023 ▸).

Di­sulfides are an important class of organic compounds with a variety of biological and pharmacological applications (Sevier & Kaiser, 2002 ▸; Lee *et al.*, 2013 ▸), in particular due to their anti­oxidant and prooxidant properties (Zhu *et al.*, 2023 ▸). It is well known that the dibromine and dichlorine oxidation of di­aryl­disulfides leads to the cleavage of the sulfur–sulfur bond (Zincke reaction; Zincke,1911 ▸; Baker *et al.*, 1946 ▸), whereas the reaction of di­sulfides with the mildest oxidant, diiodine, does not involve the cleavage of the S—S bond (Aragoni *et al.*, 2023 ▸). The reaction of 2,2′-di­pyridyl­disulfide (**L**) with I_2_ in CH_2_Cl_2_ afforded the compound [(H**L** ^+^)(I^−^)·5/2I_2_]_∞_, featuring an unusual polyiodide network counterbalancing the N-monoprotonated H**L** ^+^ cation. Recently, an assembly isostructural to [(H**L** ^+^)(I^−^)·5/2I_2_]_∞_ was obtained by reacting 2,2′-di­pyridyl­diselenide with I_2_ in either CH_2_Cl_2_ or CH_3_CN (Aragoni *et al.*, 2023 ▸).

Although 4,4′-di­pyridyl­disulfide (**L′**) has been widely reported as a donor towards a variety of metal ions (Sarkar *et al.*, 2016 ▸; Zheng *et al.*, 2022 ▸, 2023 ▸; Singha *et al.*, 2018 ▸), its reactivity towards halogens or inter­halogens has been only marginally explored (Wzgarda-Raj *et al.*, 2021 ▸; Coe *et al.*, 1997 ▸). An example is provided by 4,4′-(disulfanedi­yl)dipyridinium penta­iodide triiodide (CSD code OXAFIF; Wzgarda-Raj *et al.*, 2021 ▸) where the cation H_2_ **L′** ^2+^ is counterbalanced by a polyiodide built up of inter­acting I_3_^−^ and I_5_^−^ ions.

Following our investigation on the reactivity of polypyridyl substrates towards ICl (Aragoni *et al.*, 2008 ▸), we report here on the structural and spectroscopic characterization of the novel salt 4,4′-disulfanediyldipyridinium chloride triiodide (**1**). 
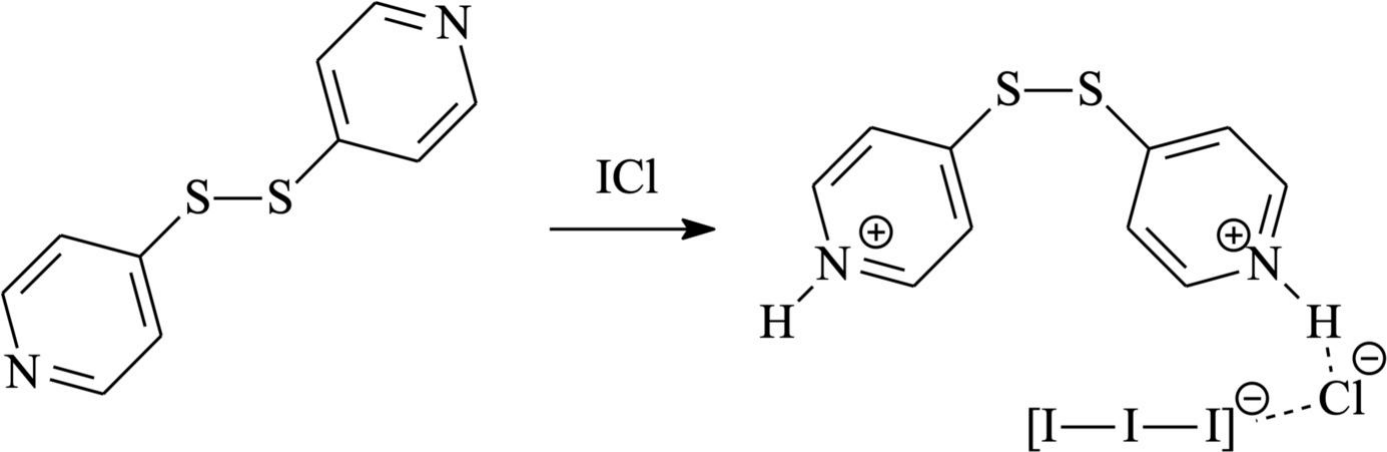


## 2. Structural commentary

**Figure 1:**
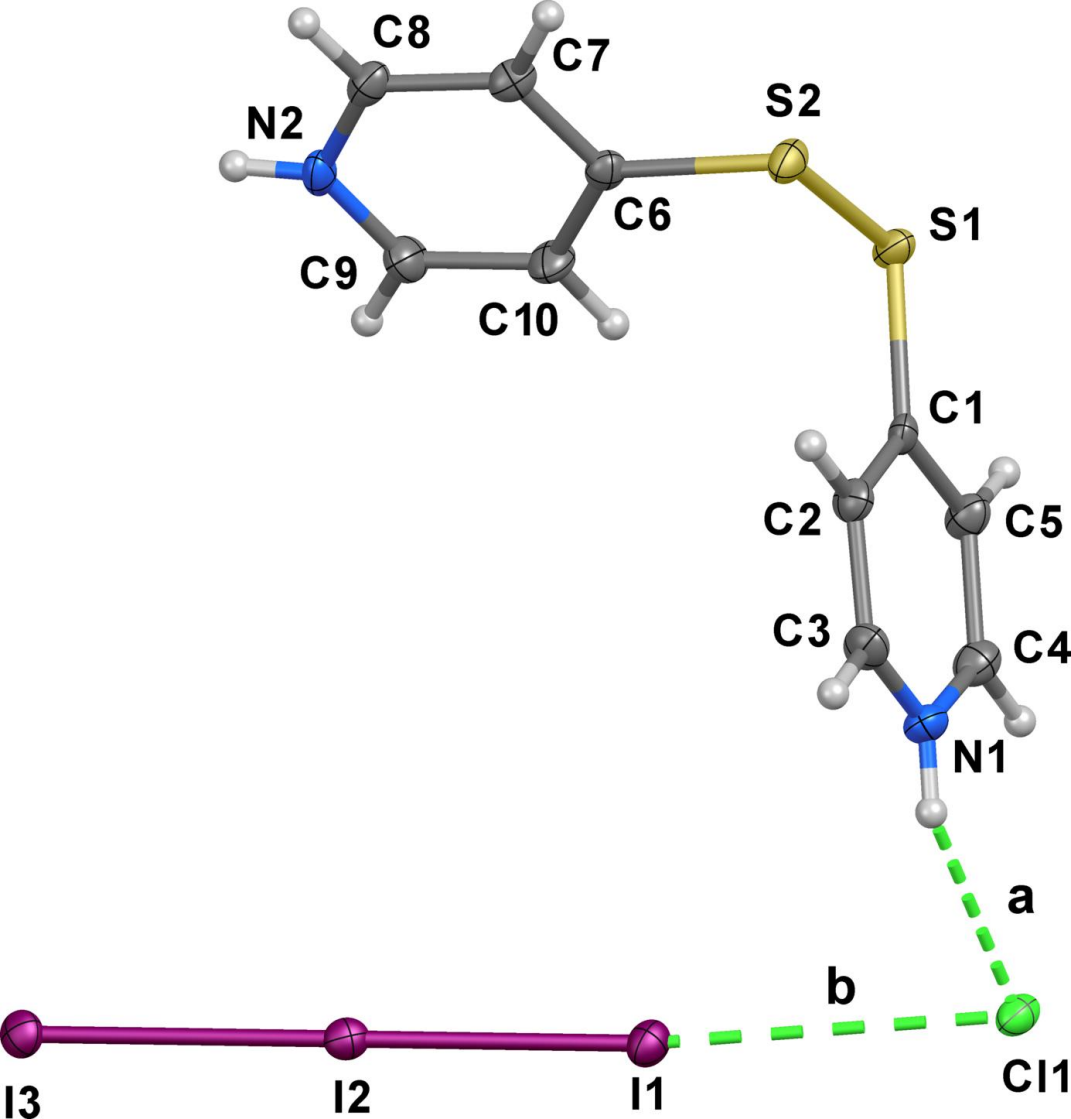
Ellipsoid plot of compound **1** with the numbering scheme adopted. Displacement ellipsoids are drawn at the 50% probability level. Labelled inter­actions are described according to Table 1 ▸.

By reacting 4,4′-di­pyridyl­disulfide (**L′**) and ICl in 1:1 molar ratio, product **1** was isolated and characterized by elemental analysis, melting point determination, FT–IR, and FT–Raman spectroscopy. Single-crystal X-ray diffraction analysis established **1** as (H_2_ **L′** ^2+^)(Cl^−^)(I_3_^−^) (Fig. 1 ▸).

Compound **1** crystallizes in the monoclinic space group *P*2_1_/*c* with four (H_2_ **L′** ^2+^)(Cl^−^)(I_3_^−^) units in the unit cell. The asymmetric unit of compound **1** consists of a donor mol­ecule protonated at both the N1 and N2 pyridine nitro­gen atoms H_2_ **L′** ^2+^ counterbalanced by a chloride and a triiodide I_3_^−^ anions. In the H_2_ **L′** ^2+^ cation, the two pyridine rings are almost perpendicular [C1—S1—S2—C6 torsion angle = 89.4 (1)°], being rotated by 2.7 (3) and 19.8 (3)° with respect to the respective C–S–S plane. The linear triiodide anion [I1—I2—I3 = 177.13 (1)°] is remarkably asymmetric with a very short I1—I2 distance [2.8180 (4) Å], close to the I—I distance of solid-state iodine (2.715 Å; van Bolhuis *et al.* 1967 ▸), and a longer one [I2—I3 = 3.0459 (4) Å], in agreement to the three-body system of the I_3_^−^ anion, showing a correlation between the two I—I distances. Accordingly, the I1—I2 and I2—I3 bond distances fall in the correlation reported by Devillanova (Aragoni *et al.*, 2012 ▸) featured by I_A_–I_B_–I_C_ systems, which correlates the relative elongations of the two I_A_—I_B_ and I_C_—I_D_ lengths with respect to the the sum of the relevant covalent radii.

## 3. Supra­molecular features

**Figure 2:**
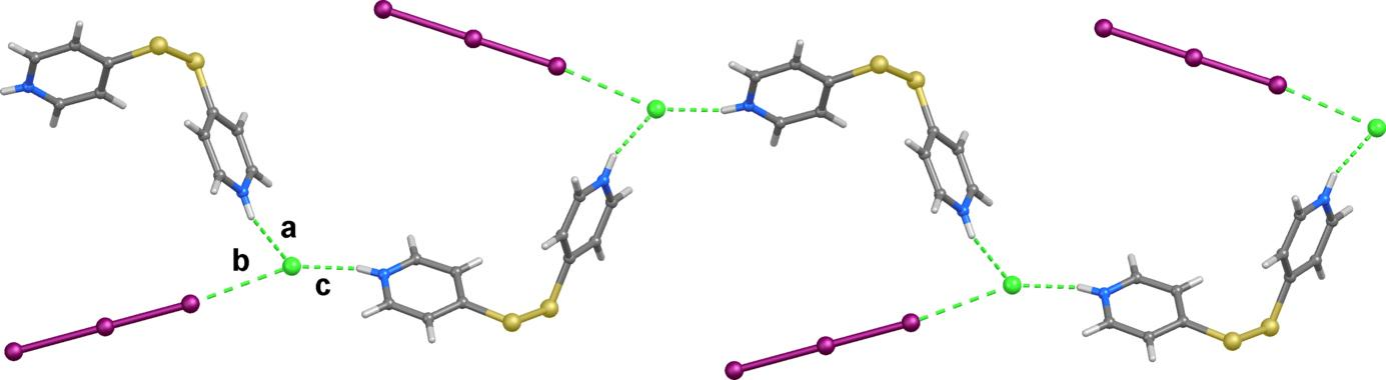
Section of the crystal packing of compound **1** viewed along the *c*-axis. Labelled contacts are described in Table 1 ▸.

The protonated pyridine rings of the H_2_ **L′** ^2+^ cation are involved in hydrogen-bonding (HB) inter­actions with the chloride anions (inter­action *a* in Fig. 1 ▸; *a* and *c* in Fig. 2 ▸ and Table 1 ▸), thus forming a wavy 1-D hydrogen-bonded polymeric structure that develops perpendicular to the *b*-axis. In addition, each chloride inter­acts with a terminal iodine atom of a triiodide [I1⋯Cl1 = 3.4764 (8) Å; inter­action *b* in Figs. 1 ▸ and 2 ▸ and Table 1 ▸] at a distance shorter than the sum of the relevant van der Waals radii (3.73 Å; Bondi, 1964 ▸), so that the chloride and the triiodide could be considered to form a [I⋯I–I⋯Cl]^2–^ dianionic ensemble, unprecedented among the relevant polyinter­halides (Sonnenberg *et al.*, 2020 ▸) deposited at the Cambridge Structural Database (CSD, version 5.45 update 1, March 2024; Groom *et al.*, 2016 ▸). Nevertheless, the Cl⋯I distance is longer than those previously reported for the parent [I_2_Cl]^−^ anion [for example I⋯Cl = 3.158, 3.047 Å in the structures with CSD codes BEQXEA (Wang *et al.* 1999 ▸) and BOJYIL (Pan *et al.* 2019 ▸), respectively] and [Cl_2_I_2_]^2–^ dianions [3.070 and 3.242 Å in DOXDOL (Buist & Kennedy, 2014 ▸) and JUPCAA (Pan *et al.* 2015 ▸), respectively]. These Cl⋯I inter­actions, shown in Fig. 2 ▸, which fall into the realm of halogen bonding (XB) inter­actions, generate the crystal packing along with a set of weak C—H⋯I contacts (entries *d*–*g* in Table 1 ▸).

## 4. Conclusions

4,4′-Disulfanediyldipyridinium chloride triiodide (H_2_ **L′** ^2+^)(Cl^−^)(I_3_^−^)(**1**) was synthesized and characterized structurally and spectroscopically. The isolation of **1** confirms that **L′** is not susceptible to the oxidative cleavage of the S—S di­sulfide bond by diiodine and iodine monochloride under mild conditions, but that it can undergo protonation and template fascinating supra­molecular structures, as previously observed in the case of [(H**L** ^+^)(I^−^)·5/2I_2_]_∞._ Further studies are ongoing in our laboratory to investigate the reactivity of different di­pyridyl­dichalcogenides towards dihalogens and inter­halogens and their versatility as building blocks for extended supra­molecular assemblies based on σ-hole inter­actions.

## 5. Synthesis and crystallization

### 5.1. Materials and methods

All the reagents and solvents were used without further purification. Elemental analysis determinations were performed with an EA1108 CHNS-O Fisons instrument. Fourier-Transform Infrared (FT–IR) spectroscopic measurements were recorded on a Bruker IFS55 spectrometer at room temperature using a flow of dried air. Far-infrared (FIR; 500–50 cm^−1^) spectra were recorded on polythene pellets using a Mylar beam-splitter and polythene windows (resolution 2 cm^−1^). Middle-infrared (MIR) spectra were recorded on KBr pellets, with a KBr beam-splitter and KBr windows (resolution 2 cm^−1^). FT-Raman spectroscopy measurements were recorded on a Bruker RFS100 spectrometer (resolution of 2 cm^−1^), with an In–Ga–As detector operating with a Nd:YAG laser (λ = 1064 nm) with a 180° scattering geometry (excitation power 5 mW). Melting point determinations were carried out on a FALC mod. C apparatus.

### 5.2. Synthesis of compound 1

To 2 mL of a CH_2_Cl_2_ solution of 4,4′-di­pyridyl­disulfide (19 mg, 8.6·10^−5^ mol), a 0.054 mol L^−1^ solution of ICl in the same solvent was added dropwise in donor/ICl in a 1:1 molar ratio. A brown crystalline precipitate was isolated from the mother liquor by air-evaporation and washed with light petroleum ether. A small number of crystals were placed on a glass slide and coated with a perfluoro­ether oil. A crystal suitable for X-ray diffraction analysis was selected and mounted on a glass fibre. Elemental analysis calculated for C_10_H_10_N_2_S_2_I_3_Cl: 18.81; H, 1.57; N, 4.38; S, 10.04%. Found: C, 18.63; H, 1.78; N, 4.09, S 9.98%. M.p. > 513 K. FT–MIR (KBr pellet, 4000–400 cm^−1^): 3854*s*, 3460*s*, 3437*s*, 3088*s*, 2743*s*, 2363*s*, 1952*s*, 1846*s*, 1773*m*, 1653*s*, 1603*s*, 1589*s*, 1558*m*, 1441*s*, 1371*s*, 1277*s*, 1086*m*, 1034*m*, 997*m*, 951*m*, 783*m*, 773*s*, 617*s*, 498*m* cm^−1^. FT–FIR (polythene pellet, 500–50 cm^­–1^): 484*m*, 477*m*, 449*w*, 418*m*, 390*w*, 378*m*, 352*m*, 294*w*, 256*m*, 227*s*, 170*s*, 131*m*, 94*m*, 67*m* cm^−1^. FT–Raman (500–50 cm^−1^, 5 mW, relative intensities in parentheses related to the highest peak taken equal to 10.0): 267(0.7), 155 (2.2), 137 (3.0), 113 (10.0) cm^−1^.

## 6. Refinement

Crystal data, data collection and structure refinement details are summarized in Table 2 ▸. H atoms bonded to heteroatoms could be located from difference-Fourier maps and their positions were freely refined. Other H atoms were placed in geometrically calculated positions and were constrained to ride on their parent atom with C—H = 0.95 Å and with *U*_iso_(H) = 1.2*U*_eq_(C).

## Supplementary Material

Crystal structure: contains datablock(s) I. DOI: 10.1107/S2056989024004213/ee2006sup1.cif

Structure factors: contains datablock(s) I. DOI: 10.1107/S2056989024004213/ee2006Isup2.hkl

Supporting information file. DOI: 10.1107/S2056989024004213/ee2006Isup3.mol

Supporting information file. DOI: 10.1107/S2056989024004213/ee2006Isup4.cml

CCDC reference: 2330302

Additional supporting information:  crystallographic information; 3D view; checkCIF report

## Figures and Tables

**Table 1:** Inter­molecular inter­actions (Å, °) of compound **1**

Inter­action		A—B	B⋯C	A⋯C	A—B⋯C
*a*	N1—H1⋯Cl1	0.82 (3)	2.41 (3)	3.101 (2)	142 (2)
*b*	I2—I1⋯Cl1	2.8179 (4)	3.4764 (8)	–	173.93 (2)
*c*	N2^i^—H2^i^⋯Cl1	0.81 (4)	2.21 (4)	3.006 (3)	168
*d*	C10—H10⋯I3^ii^	0.95	3.07	3.833 (3)	138
*e*	C9—H9⋯I2^ii^	0.95	3.18	4.108 (4)	166
*f*	C7—H7⋯I2^iii^	0.95	3.03	3.738 (4)	132
*g*	C7—H7⋯I3^iii^	0.95	3.14	3.801 (3)	129

Symmetry codes: (i) −1 + *x*, 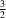 − *y*, − 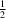 + *z*; (ii) 2 − *x*, 2 − *y*, 1 − *z*; (iii) *x*, 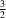 − *y*, 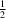 + *z*.

**Table 2:** Experimental details

Crystal data	
Chemical formula	C_10_H_10_N_2_S_2_^2+^·Cl^−^·I_3_^−^
*M*_r_	638.47
Crystal system, space group	Monoclinic, *P*2_1_/*c*
Temperature (K)	120
*a*, *b*, *c* (Å)	12.9631 (11), 11.3802 (5), 13.1675 (11)
β (°)	117.624 (6)
*V* (Å^3^)	1721.1 (2)
*Z*	4
Radiation type	Mo *K*α
μ (mm^−1^)	5.83
Crystal size (mm)	0.32 × 0.22 × 0.06

Data collection	
Diffractometer	Bruker-Nonius 95mm CCD camera on κ-goniostat
Absorption correction	Multi-scan (*SADABS*; Krause *et al.*, 2015 ▸)
*T*_min_, *T*_max_	0.632, 1.000
No. of measured, independent and observed [*I* > 2σ(*I*)] reflections	16769, 3959, 3621
*R*_int_	0.027
(sin θ/λ)_max_ (Å^−1^)	0.651

Refinement	
*R*[*F* ^2^ > 2σ(*F* ^2^)], *wR*(*F* ^2^), *S*	0.022, 0.046, 1.13
No. of reflections	3959
No. of parameters	170
H-atom treatment	H atoms treated by a mixture of independent and constrained refinement
Δρ_max_, Δρ_min_ (e Å^−3^)	0.74, −0.80

Computer programs: *DENZO* (Otwinowski & Minor, 1997 ▸) & *COLLECT* (Hooft, 1998 ▸), *SHELXT2018/2* (Sheldrick, 2015*a* ▸), *SHELXL2018/3* (Sheldrick, 2015*b* ▸) and *OLEX2* (Dolomanov *et al.*, 2009 ▸).

## supplementary crystallographic information

### Crystal data

**Table:**

C_10_H_10_N_2_S_2_^2+^·Cl^−^·I_3_^−^	*F*(000) = 1168
*M**_r_* = 638.47	*D*_x_ = 2.464 Mg m^−^^3^
Monoclinic, *P*2_1_/*c*	Mo *K*α radiation, λ = 0.71073 Å
*a* = 12.9631 (11) Å	Cell parameters from 17448 reflections
*b* = 11.3802 (5) Å	θ = 2.9–27.5°
*c* = 13.1675 (11) Å	µ = 5.83 mm^−^^1^
β = 117.624 (6)°	*T* = 120 K
*V* = 1721.1 (2) Å^3^	Cut-plate, brown
*Z* = 4	0.32 × 0.22 × 0.06 mm

### Data collection

**Table:**

Bruker-Nonius 95mm CCD camera on κ-goniostat diffractometer	3621 reflections with *I* > 2σ(*I*)
Detector resolution: 9.091 pixels mm^-1^	*R*_int_ = 0.027
φ & ω scans	θ_max_ = 27.6°, θ_min_ = 3.1°
Absorption correction: multi-scan (SADABS; Krause *et al.*, 2015)	*h* = −16→16
*T*_min_ = 0.632, *T*_max_ = 1.000	*k* = −14→14
16769 measured reflections	*l* = −16→17
3959 independent reflections	

### Refinement

**Table:**

Refinement on *F*^2^	Hydrogen site location: mixed
Least-squares matrix: full	H atoms treated by a mixture of independent and constrained refinement
*R*[*F*^2^ > 2σ(*F*^2^)] = 0.022	*w* = 1/[σ^2^(*F*_o_^2^) + (0.0127*P*)^2^ + 2.0409*P*] where *P* = (*F*_o_^2^ + 2*F*_c_^2^)/3
*wR*(*F*^2^) = 0.046	(Δ/σ)_max_ = 0.003
*S* = 1.13	Δρ_max_ = 0.74 e Å^−^^3^
3959 reflections	Δρ_min_ = −0.80 e Å^−^^3^
170 parameters	Extinction correction: *SHELXL2018/3* (Sheldrick, 2015b), Fc^*^=kFc[1+0.001xFc^2^λ^3^/sin(2θ)]^-1/4^
0 restraints	Extinction coefficient: 0.00091 (7)
Primary atom site location: dual	

### Special details

**Table:**

Geometry. All esds (except the esd in the dihedral angle between two l.s. planes) are estimated using the full covariance matrix. The cell esds are taken into account individually in the estimation of esds in distances, angles and torsion angles; correlations between esds in cell parameters are only used when they are defined by crystal symmetry. An approximate (isotropic) treatment of cell esds is used for estimating esds involving l.s. planes.

### Fractional atomic coordinates and isotropic or equivalent isotropic displacement parameters (Å^2^)

**Table:**

	*x*	*y*	*z*	*U*_iso_*/*U*_eq_	
I1	0.67827 (2)	1.09634 (2)	0.41894 (2)	0.01952 (7)	
I2	0.91260 (2)	1.16703 (2)	0.50892 (2)	0.01704 (6)	
I3	1.16454 (2)	1.24311 (2)	0.59403 (2)	0.01907 (7)	
Cl1	0.39992 (6)	0.98349 (6)	0.32707 (6)	0.01852 (15)	
S2	0.78491 (6)	0.37523 (6)	0.66898 (6)	0.01882 (16)	
S1	0.68199 (6)	0.41735 (6)	0.50222 (6)	0.01885 (16)	
N2	1.1441 (2)	0.4996 (2)	0.7561 (2)	0.0192 (5)	
H2	1.211 (3)	0.515 (3)	0.773 (3)	0.023*	
N1	0.5417 (2)	0.7795 (2)	0.4871 (2)	0.0199 (5)	
H1	0.516 (3)	0.847 (3)	0.477 (3)	0.024*	
C6	0.9235 (2)	0.4272 (2)	0.6967 (2)	0.0158 (6)	
C7	1.0175 (3)	0.3800 (3)	0.7912 (2)	0.0177 (6)	
H7	1.005658	0.321243	0.836033	0.021*	
C8	1.1276 (3)	0.4183 (3)	0.8198 (2)	0.0184 (6)	
H8	1.192339	0.386896	0.885121	0.022*	
C10	0.9433 (3)	0.5117 (3)	0.6312 (3)	0.0218 (6)	
H10	0.880332	0.545123	0.565665	0.026*	
C9	1.0561 (3)	0.5457 (3)	0.6637 (3)	0.0221 (6)	
H9	1.071345	0.602813	0.619735	0.027*	
C1	0.6298 (2)	0.5595 (2)	0.5052 (2)	0.0152 (6)	
C5	0.5599 (3)	0.6079 (3)	0.3976 (3)	0.0198 (6)	
H5	0.542253	0.564513	0.329894	0.024*	
C2	0.6521 (2)	0.6235 (3)	0.6030 (2)	0.0180 (6)	
H2A	0.698461	0.591158	0.676964	0.022*	
C3	0.6063 (3)	0.7340 (3)	0.5911 (3)	0.0223 (7)	
H3	0.620692	0.778684	0.657258	0.027*	
C4	0.5170 (3)	0.7196 (3)	0.3912 (3)	0.0209 (6)	
H4	0.469740	0.754210	0.318565	0.025*	

### Atomic displacement parameters (Å^2^)

**Table:**

	*U*^11^	*U*^22^	*U*^33^	*U*^12^	*U*^13^	*U*^23^
I1	0.01655 (11)	0.02240 (11)	0.02038 (11)	0.00155 (7)	0.00921 (9)	0.00205 (8)
I2	0.01911 (11)	0.01738 (10)	0.01659 (11)	0.00135 (7)	0.00995 (8)	0.00154 (7)
I3	0.01743 (11)	0.02150 (11)	0.01500 (11)	−0.00096 (7)	0.00473 (8)	−0.00102 (7)
Cl1	0.0157 (3)	0.0192 (3)	0.0216 (4)	0.0040 (3)	0.0095 (3)	0.0045 (3)
S2	0.0134 (4)	0.0209 (3)	0.0225 (4)	0.0021 (3)	0.0086 (3)	0.0052 (3)
S1	0.0150 (4)	0.0173 (3)	0.0196 (4)	0.0037 (3)	0.0042 (3)	−0.0033 (3)
N2	0.0117 (12)	0.0193 (12)	0.0274 (14)	−0.0021 (10)	0.0098 (11)	−0.0063 (11)
N1	0.0167 (13)	0.0164 (12)	0.0264 (14)	0.0037 (10)	0.0099 (11)	0.0005 (11)
C6	0.0150 (14)	0.0162 (13)	0.0173 (14)	0.0015 (11)	0.0083 (12)	−0.0029 (11)
C7	0.0193 (15)	0.0203 (14)	0.0148 (14)	0.0041 (12)	0.0091 (12)	0.0013 (12)
C8	0.0148 (15)	0.0221 (14)	0.0165 (14)	0.0033 (11)	0.0056 (12)	−0.0029 (12)
C10	0.0186 (16)	0.0207 (15)	0.0225 (15)	0.0042 (12)	0.0064 (13)	0.0084 (12)
C9	0.0205 (16)	0.0199 (14)	0.0282 (17)	−0.0015 (12)	0.0131 (14)	0.0028 (13)
C1	0.0084 (13)	0.0163 (13)	0.0187 (14)	−0.0010 (10)	0.0043 (11)	−0.0023 (11)
C5	0.0175 (15)	0.0221 (15)	0.0177 (15)	0.0029 (12)	0.0064 (12)	−0.0027 (12)
C2	0.0153 (15)	0.0191 (14)	0.0147 (14)	0.0007 (11)	0.0027 (12)	0.0000 (12)
C3	0.0200 (16)	0.0199 (14)	0.0229 (16)	−0.0006 (12)	0.0065 (13)	−0.0079 (13)
C4	0.0194 (16)	0.0228 (15)	0.0207 (15)	0.0036 (12)	0.0094 (13)	0.0043 (13)

### Geometric parameters (Å, º)

**Table:**

I1—I2	2.8180 (4)	C7—C8	1.368 (4)
I2—I3	3.0459 (4)	C8—H8	0.9500
S2—S1	2.0285 (11)	C10—H10	0.9500
S2—C6	1.762 (3)	C10—C9	1.375 (4)
S1—C1	1.762 (3)	C9—H9	0.9500
N2—H2	0.81 (3)	C1—C5	1.394 (4)
N2—C8	1.331 (4)	C1—C2	1.387 (4)
N2—C9	1.330 (4)	C5—H5	0.9500
N1—H1	0.82 (3)	C5—C4	1.374 (4)
N1—C3	1.334 (4)	C2—H2A	0.9500
N1—C4	1.337 (4)	C2—C3	1.368 (4)
C6—C7	1.384 (4)	C3—H3	0.9500
C6—C10	1.393 (4)	C4—H4	0.9500
C7—H7	0.9500		
			
I1—I2—I3	177.129 (8)	C9—C10—H10	120.8
C6—S2—S1	103.93 (10)	N2—C9—C10	120.7 (3)
C1—S1—S2	105.03 (10)	N2—C9—H9	119.6
C8—N2—H2	116 (2)	C10—C9—H9	119.6
C9—N2—H2	122 (2)	C5—C1—S1	114.5 (2)
C9—N2—C8	122.0 (3)	C2—C1—S1	125.9 (2)
C3—N1—H1	123 (2)	C2—C1—C5	119.6 (3)
C3—N1—C4	122.1 (3)	C1—C5—H5	120.6
C4—N1—H1	115 (2)	C4—C5—C1	118.8 (3)
C7—C6—S2	116.4 (2)	C4—C5—H5	120.6
C7—C6—C10	119.1 (3)	C1—C2—H2A	120.6
C10—C6—S2	124.5 (2)	C3—C2—C1	118.9 (3)
C6—C7—H7	120.2	C3—C2—H2A	120.6
C8—C7—C6	119.6 (3)	N1—C3—C2	120.5 (3)
C8—C7—H7	120.2	N1—C3—H3	119.8
N2—C8—C7	120.0 (3)	C2—C3—H3	119.8
N2—C8—H8	120.0	N1—C4—C5	120.1 (3)
C7—C8—H8	120.0	N1—C4—H4	120.0
C6—C10—H10	120.8	C5—C4—H4	120.0
C9—C10—C6	118.4 (3)		
			
S2—S1—C1—C5	177.6 (2)	C7—C6—C10—C9	−0.4 (4)
S2—S1—C1—C2	−2.7 (3)	C8—N2—C9—C10	0.9 (4)
S2—C6—C7—C8	−178.3 (2)	C10—C6—C7—C8	1.1 (4)
S2—C6—C10—C9	178.9 (2)	C9—N2—C8—C7	−0.2 (4)
S1—S2—C6—C7	−160.8 (2)	C1—C5—C4—N1	−0.5 (4)
S1—S2—C6—C10	19.9 (3)	C1—C2—C3—N1	−0.2 (4)
S1—C1—C5—C4	−178.7 (2)	C5—C1—C2—C3	−1.2 (4)
S1—C1—C2—C3	179.1 (2)	C2—C1—C5—C4	1.5 (4)
C6—C7—C8—N2	−0.8 (4)	C3—N1—C4—C5	−0.9 (5)
C6—C10—C9—N2	−0.5 (4)	C4—N1—C3—C2	1.3 (5)
